# A Computational Biomarker of Photosensitive Epilepsy from Interictal EEG

**DOI:** 10.1523/ENEURO.0486-21.2022

**Published:** 2022-06-17

**Authors:** Marinho A. Lopes, Sanchita Bhatia, Glen Brimble, Jiaxiang Zhang, Khalid Hamandi

**Affiliations:** 1 Cardiff University Brain Research Imaging Centre, School of Psychology, Cardiff University, Cardiff CF24 4HQ, United Kingdom; 2 Department of Clinical Neurophysiology, University Hospital of Wales, Cardiff CF14 4XW, United Kingdom; 3 The Welsh Epilepsy Unit, Department of Neurology, University Hospital of Wales, Cardiff CF14 4XW, United Kingdom

**Keywords:** functional network, hyperexcitability, interictal EEG, mathematical model, photosensitive epilepsy

## Abstract

People with photosensitive epilepsy (PSE) are prone to seizures elicited by visual stimuli. The possibility of inducing epileptiform activity in a reliable way makes PSE a useful model to understand epilepsy, with potential applications for the development of new diagnostic methods and new treatments for epilepsy. A relationship has been demonstrated between PSE and both occipital and more widespread cortical hyperexcitability using various types of stimulation. Here we aimed to test whether hyperexcitability could be inferred from resting interictal electroencephalographic (EEG) data without stimulation. We considered a cohort of 46 individuals with idiopathic generalized epilepsy who underwent EEG during intermittent photic stimulation: 26 had a photoparoxysmal response (PPR), the PPR group, and 20 did not, the non-PPR group. For each individual, we computed functional networks from the resting EEG data before stimulation. We then placed a computer model of ictogenicity into the networks and simulated the propensity of the network to generate seizures *in silico* [the brain network ictogenicity (BNI)]. Furthermore, we computed the node ictogenicity (NI), a measure of how much each brain region contributes to the overall ictogenic propensity. We used the BNI and NI as proxies for testing widespread and occipital hyperexcitability, respectively. We found that the BNI was not higher in the PPR group relative to the non-PPR group. However, we observed that the (right) occipital NI was significantly higher in the PPR group relative to the non-PPR group. Other regions did not have significant differences in NI values between groups.

## Significance Statement

We used a computational framework to assess widespread and occipital hyperexcitability in people with epilepsy from apparently normal EEG results. We aimed at distinguishing individuals with photosensitivity from individuals without this susceptibility to seizures provoked by visual stimuli. Our results suggest that either widespread hyperexcitability did not differ between the two groups of individuals, or that our methods were not appropriate to measure this hyperexcitability. Conversely, we observed higher occipital hyperexcitability in the photosensitive group compared with the other group. This finding suggests that occipital hyperexcitability is an enduring feature in the brain activity of people with photosensitivity. Thus, our results suggest that our methods based on resting-state EEG may aid the diagnosis of photosensitive epilepsy without requiring stimulation.

## Introduction

People with epilepsy are prone to unprovoked epileptic seizures (Fisher et al., 2005). A subset of individuals also has seizures triggered by stimuli. The most common reflex epilepsy is photosensitive epilepsy (PSE), which occurs in ∼2–5% of people with epilepsy (Padmanaban et al., 2019). In addition to seizures, visual stimuli can also induce EEG epileptiform activity in PSE. Thus, PSE can be diagnosed by recording a photoparoxysmal response (PPR) in an electroencephalogram (EEG) during intermittent photic stimulation (IPS). PSE is commonly seen in the idiopathic generalized epilepsies (IGEs), in particular, juvenile myoclonic epilepsy (JME), ranging from 30% to 90% (Wolf and Goosses, 1986; Appleton et al., 2000). PSE can serve as a useful model to understand epilepsy (Padmanaban et al., 2019). The PPR phenotype has been used within clinical trials to test the efficacy of antiseizure medication (Yuen and Sims, 2014) and in assessing other neurophysiological responses to visual stimuli (Parra et al., 2003; Perry et al., 2014). Thus, a better understanding of the pathophysiology of PSE may have an impact on the diagnosis and treatments of epilepsy.

Various studies have found evidence for both occipital and more widespread cortical hyperexcitability in people with PSE (Padmanaban et al., 2019). By using a paired‐pulse flash‐evoked technique, Strigaro et al. (2012) showed decreased inhibition in the visual system of people with PSE. Siniatchkin et al. (2007) used transcranial magnetic stimulation (TMS) to investigate the excitability of the visual and primary motor cortices in people with and without PPR. They observed that people with PPR have a significantly lower phosphene threshold (i.e., a lower TMS amplitude is required to elicit a perception of light in people with PPR). They also found that occipital TMS can more easily suppress visual perception in people with PPR than in those without it (Siniatchkin et al., 2007). Brigo et al. (2013) have also used TMS in PSE and provided further supporting evidence for these observations. Beyond the occipital lobe, there is evidence of the involvement of motor areas. Moeller et al. (2009, 2013) have used EEG combined with functional magnetic resonance imaging (fMRI) to show increased blood oxygenation level-dependent (BOLD) signal within the visual cortex, and the premotor and parietal cortices after a PPR has been provoked by IPS. By using magnetoencephalography, Parra et al. (2003) observed enhanced network-level synchrony within the gamma band just before PPR formation. Vaudano et al. (2017) considered resting-state EEG-fMRI and found a decreased alpha-related inhibition of the visual cortex and sensory–motor networks in people with PSE versus other individuals with epilepsy (i.e., a lower anticorrelation between EEG alpha power and the BOLD signal in those networks). Diffusion tensor imaging studies have also revealed structural connectivity abnormalities within motor areas and the occipital lobe in individuals with PSE in IGE and JME, which may underpin hyperexcitability in those regions (Groppa et al., 2012; Vollmar et al., 2012).

Since a propensity for ictogenicity can be a consequence of hyperexcitability, here we aimed to find out whether widespread and/or occipital increases in the propensity for ictogenicity may be identified from resting interictal EEGs in people with PSE. As a proxy to evaluate network-wide and local propensity for ictogenicity, we used the brain network ictogenicity (BNI) and node ictogenicity (NI), respectively. BNI is a measure of how likely a functional brain network is to generate seizure-like activity in computer simulations (Petkov et al., 2014; Lopes et al., 2017). These simulations consist in placing a mathematical model of normal and abnormal epileptic EEG features into the functional network and computing the resulting brain dynamics. Functional networks more prone to seizures are expected to produce more seizure-like activity in the simulations (Petkov et al., 2014; Lopes et al., 2017, 2021). Node ictogenicity is assessed by removing regions from the functional network and evaluating the resulting altered BNI (Goodfellow et al., 2016; Lopes et al., 2017). Brain regions whose removal produces a higher reduction of BNI are considered more likely to drive seizures.

We studied retrospective EEG recordings from two groups of individuals with IGE, one that had PPR during IPS (the PPR group), and another that did not have PPR (the non-PPR group). We tested the following two hypotheses: (1) the PPR group has a higher BNI than the non-PPR group; and (2) the PPR group has a higher occipital NI than the non-PPR group. We aimed to test whether interictal EEG may be used to predict PSE and whether the mechanisms of PPR are enduring features present in interictal brain states even without stimulation.

## Materials and Methods

### Data

We searched EEG reports at the University Hospital of Wales (Cardiff, UK) from 2007 to 2017 using the terms, “photoparoxysmal response,” “PPR,” “IGE,” “JME,” “JAE” (juvenile absence epilepsy), and childhood absence epilepsy “CAE,” and limited our search to individuals who were 12–32 years of age at the time of EEG. Clinical EEG reports were reviewed to identify two cohorts of individuals with (1) IGE with IPS and PPR, and (2) IGE with IPS and no PPR. The IPS protocol at our center followed the international standard recommendations set out in the International League Against Epilepsy guidelines (Kasteleijn-Nolst Trenité et al., 2012). This comprised 10 s of IPS, 5 s with eyes open and 5 s with eyes closed, at the following incrementally increasing and then decreasing frequencies (1, 2, 4, 6, 8, 10, 12, 14, 16, 18, 20, 60, 50, 40, 30, 25, and 20 Hz). The IPS was immediately stopped if a PPR was observed (i.e., a spike-wave discharge induced by the light stimulation). In some individuals, the order of stimulation was varied to look for reproducible PPR or to cease IPS if frequent PPR was already seen. We selected 29 individuals with PPR seen on EEGs and 20 individuals in the group without PPR. Henceforth, we will refer to the two groups as the PPR group and the non-PPR group, respectively.

**Figure 1.**
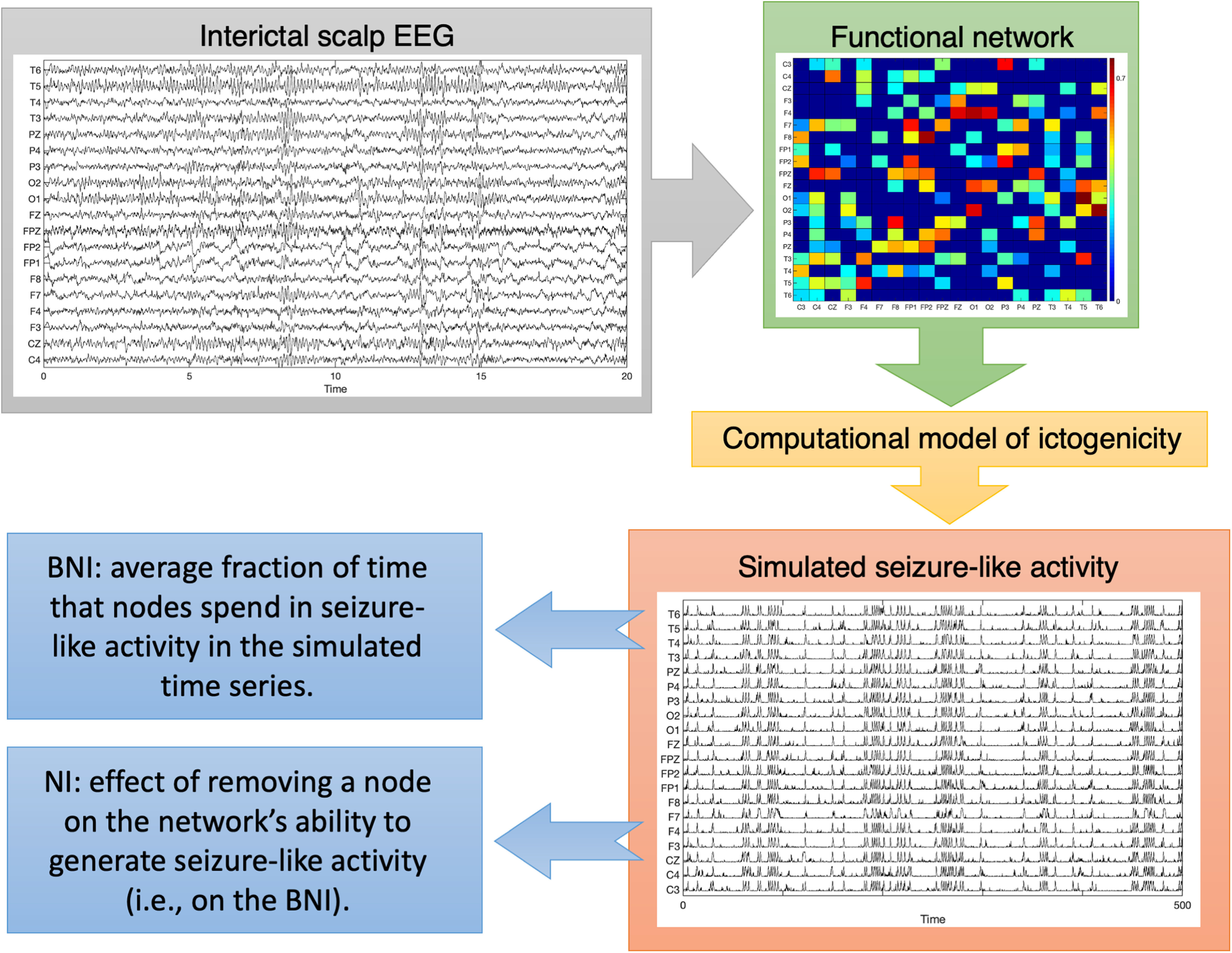
Summary of our computational method. We used interictal scalp EEG to infer functional networks. Then, to interrogate the networks, we considered a computational model of ictogenicity to simulate seizure-like activity on the functional networks. This allowed us to assess the propensity of a functional network to generate seizures *in silico*, the BNI. It further enabled us to measure the NI (i.e., the effect of removing a node on the BNI), which represents the local ictogenic propensity.

The routine clinical EEG included ∼20 min of resting-state interictal EEG preceding the IPS procedure. To test whether we could predict the individuals’ responses based on the interictal EEG recorded before IPS, we selected three continuous segments of 20 s per individual within the 20 min window. The selection criteria were such as to avoid distinct (eye movement, muscle) artifacts and epileptiform activity, and such that all three segments were at least 1 min apart from each other. Three individuals in the PPR group were excluded from this study because we could not find interictal segments without marked artifacts within their EEG sessions. Thus, we studied a total of 46 individuals (26 PPR individuals and 20 non-PPR individuals). Tables 1 and 2 show the demographics and clinical information of these two groups. Figure 1 provides an example of a 20 s segment.

**Table 1:** Demographics and clinical information of the group of individuals that presented PPR on EEG

ID	Age (years)	Gender	Syndrome	Medication
PPR1	24	M	JME	Lamotrigine
PPR2	16	M	GTCSO	None
PPR3	15	M	GTSO	Valproate
PPR4	13	F	JAE	None
PPR5	15	F	JAE	Levetiracetam, lamotrigine
PPR6	13	F	JME	None
PPR7	16	F	JME	Lamotrigine
PPR8	17	F	JME	Lamotrigine
PPR9	19	F	JAE	Clobazam
PPR10	18	M	GTCSO	None
PPR11	16	F	GTCSO	Levetiracetam
PPR12	17	F	JME	Levetiracetam
PPR13	13	F	JME	None
PPR14	24	F	JME	Valproate
PPR15	19	F	GTCSO	None
PPR16	22	F	GTCSO	None
PPR17	14	F	JME	None
PPR18	31	F	JME	None
PPR19	18	M	JME	None
PPR20	23	F	GTCSO	Levetiracetam
PPR21	19	M	GTCSO	Valproate
PPR22	23	F	GTCSO	Sertraline
PPR23	26	M	JME	Valproate, zonisamide
PPR24	13	F	JME	None
PPR25	14	F	GTCSO	None
PPR26	13	F	JAE	None

M, Male; F, female; GTCSO, generalized tonic-clonic seizures only.

**Table 2:** Demographics and clinical information of the group of individuals that did not show a PPR on EEG

ID	Age (years)	Gender	Syndrome	Medication
Non-PPR1	16	M	JME	None
Non-PPR2	28	F	JME	Valproate
Non-PPR3	30	M	JME	Valproate
Non-PPR4	19	M	JME	None
Non-PPR5	24	F	JME	Levetiracetam
Non-PPR6	18	M	JME	Epilim
Non-PPR7	19	M	JME	None
Non-PPR8	27	F	JME	Valproate
Non-PPR9	20	F	JME	Lamotrigine
Non-PPR10	18	M	JME	None
Non-PPR11	18	F	JME	None
Non-PPR12	22	F	JME	Duloxetine
Non-PPR13	22	F	JME	Lamotrigine, carbamazepine
Non-PPR14	23	M	JME	None
Non-PPR15	21	F	JME	Topiramate, levetiracetam, clobazam
Non-PPR16	30	M	GTCSO	Carbamazepine
Non-PPR17	20	F	JME	Epilim
Non-PPR18	20	F	JME	None
Non-PPR19	20	M	JME	Valproate
Non-PPR20	31	F	JME	Lamotrigine, Sumatriptan

M, Male; F, female; GTCSO, generalized tonic-clonic seizures only.

The EEG recordings were obtained using the 10–20 system for electrode placement, and we considered all the standard channels in our analysis (C3, C4, CZ, F3, F4, F7, F8, FP1, FP2, FPZ, FZ, O1, O2, P3, P4, PZ, T3, T4, T5, and T6).

The EEG data were recorded at a sampling rate, *f_s_*, of 500 or 512 Hz. For consistency, we downsampled the data to 250 Hz. Furthermore, we applied a bandpass filter in the low alpha frequency band, 6–9 Hz (fourth-order Butterworth filter with forward and backward filtering to minimize phase distortions). We focused on the low alpha band because previous studies have shown that functional networks inferred from this frequency band are informative for both epilepsy diagnosis and epilepsy classification (Schmidt et al., 2016; Lopes et al., 2019), as well as of reduced inhibition in PSE (Vaudano et al., 2017). The data were also rereferenced to the average of all artifact-free segments.

### Functional networks

To build functional networks from the 20 s interictal EEG segments, we followed a method based on previous studies (Schmidt et al., 2016; Lopes et al., 2019, 2020; Tait et al., 2021). We computed functional networks in the sensor space, where nodes corresponded to EEG channels. Connections between pairs of nodes *i* and *j* were inferred using the phase-locking value (PLV; Tass et al., 1998; Mormann et al., 2000), as follows:

$${\text{PLV}}_{ij}=\frac{1}{N_{t}}\left|\overset{N_{t}}{\underset{k=1}{\sum }}e^{i\text{Δ}\phi_{ij}(t_{k})}\right|,$$

where $N_{s}$ is the number of time points ( $N_{t}=5000$), and $\text{Δ}\phi_{ij}(t_{k})$ is the instantaneous phase difference between the EEG signals from channels i and j at time $t_{k}$, which was computed using the Hilbert transform. We also calculated the average phase lag $\tau_{ij}$ between pairs of signals, as follows:

$$\tau_{ij}=\text{arg}\left(\overset{N_{t}}{\underset{k=1}{\sum }}e^{i\text{Δ}\phi_{ij}(t_{k})}\right).$$

We considered nodes *i* and *j* to be connected at ${\text{PLV}}_{ij}>0$ and $\tau_{ij}>2\pi /f_{s}$ with connection weight ${\text{PLV}}_{ij}$. We neglected zero phase lag PLV (i.e., $\tau_{ij}<2\pi /f_{s}$) to avoid possibly artifactual relations because of volume conduction (Bastos and Schoffelen, 2016). Spurious connections were also rejected by comparing each possible PLV value to a set of PLV values obtained from surrogate time series (i.e., randomized time series comparable to the original time series). This comparison aims to remove connections whose PLV is because of random associations between signals and because of the finite nature of the signals. We generated 99 surrogates from the original EEG signals using the iterative amplitude-adjusted Fourier transform with 10 iterations (Schreiber and Schmitz, 1996, 2000) and calculated 99 PLV values for every pair of channels. PLV values that are >95% of the corresponding PLV values calculated from the surrogates were taken forward, representing the weights of the functional network. Thus, we constructed three functional networks per individual (i.e., one from each 20 s segment), each of them a matrix of statistically significant PLV values. Figure 1 shows an example of a functional network.

### Computational model

We used these functional networks and a computational model of epilepsy to address two questions. (1) Do the networks from the PPR group have a higher ictogenic propensity than those from the non-PPR group? (2) Does the PPR group have a higher local ictogenic propensity in the occipital lobe compared with the non-PPR group?

The computational model was used to simulate the ability of a functional network to generate seizures, thereby enabling us to assess the propensity of the brain for ictogenicity and to compare it across individuals. It also enables us to analyze the relative importance of each brain region to the overall propensity to ictogenicity (i.e., the local ictogenic propensity; Lopes et al., 2017, 2019, 2020). Our approach consisted in using a phenomenological model of ictogenicity, the theta model (Ermentrout and Kopell, 1986), together with the calculated functional networks, to simulate brain-like activity and seizure-like transitions in these networks (Lopes et al., 2017, 2019, 2020). In this model, the activity at network node *i* is represented by a phase oscillator, $\theta_{i}$, that obeys the following ordinary differential equation, as follows:

$$\dot{\theta_{i}}=\left(1-\cos\theta_{i}\right)+\left(1+\cos\theta_{i}\right)I_{i}\left(t\right),$$

where $I_{i}\left(t\right)$ is an input current to the node i at time t. This current is given by the following:

$$I_{i}\left(t\right)=I_{0}+\xi^{\left(i\right)}\left(t\right)+\frac{K}{N}\underset{i\neq j}{\sum }{\text{PLV}}_{ji}\left[1-\cos\left(\theta_{j}-\theta^{\left(s\right)}\right)\right],$$

where $I_{0}+\xi^{\left(i\right)}\left(t\right)$ is Gaussian noise, K is a global scaling factor of network interactions, and N is the number of nodes in the network ( N=20). The noise represents signals coming from brain regions outside of the considered functional networks and the term $\left[1-\cos(\theta_{j}-\theta^{\left(s\right)})\right]$ is the output of node j, representing a displacement from its resting-state phase $\theta^{\left(s\right)}$. $PLV_{ji}$ is *j*, the *i*th entry of the adjacency matrix that represents the functional network calculated above. Within the model, oscillators may transit between the following two states: a resting state characterized by fluctuations close to the stable phase $\theta^{\left(s\right)}$ if $I_{i}<0$, and a seizure-like state represented by a rotating phase if $I_{i}>0$. The transition between the two states corresponds to a saddle node on an invariant circle bifurcation at $I_{i}=0$. This oscillator model was shown to approximate a neural mass model for the purpose of studying the ictogenicity of a network (Lopes et al., 2017). Figure 1 shows an example of simulated signals using this model. We chose parameters according to previous studies (Lopes et al., 2017, 2019, 2020): $I_{0}=-1.2$ and noise SD σ=0.6. The global scaling factor K was treated as a free parameter (see the Brain network ictogenicity and node ictogenicity section).

### Brain network ictogenicity and node ictogenicity

We measured the ability of the brain network to generate seizures by using the concept of BNI. The BNI is the average fraction of computational time that the network nodes spend in the seizure-like state (Petkov et al., 2014; Lopes et al., 2017), as follows:

$$\text{BNI}=\frac{1}{N}\underset{i}{\sum }\frac{t_{sz}^{\left(i\right)}}{T},$$

where $t_{sz}^{\left(i\right)}$ is the time that node i spends in the rotating state during a total simulation time T. We used $T=4\times 10^{6}$, as in previous studies (Lopes et al., 2019, 2020; but see Lopes et al., 2017, for more details about the calculation of $t_{sz}^{\left(i\right)}$). To avoid an arbitrary choice of the free parameter K, we considered a robust redefinition of the BNI (Lopes et al., 2017, 2021) given by the following:

$$\begin{array}{c} \hat{\text{BNI}}=\overset{K_{2}}{\underset{K_{1}}{\int }}\text{BNI}\left(K\right)dK, \end{array}$$

where $K_{1}$ and $K_{2}$ were chosen so that to capture the full variation of the BNI from 0 to 1 for all networks under consideration. We used the same interval $\left[K_{1},K_{2}\right]=[1,40]$ for all functional networks of all individuals. For each individual, we obtained three $\hat{\text{BNI}}$ values, one per each functional network, and took their mean value for the statistical analysis below.

To quantify the relative importance of each node to the ability of the network to generate seizures (i.e., the local ictogenicity, we considered the NI; Goodfellow et al., 2016; Lopes et al., 2017, 2019). The ${\text{NI}}^{\left(i\right)}$ measures the effect of removing node i on the ability of the network to generate seizures *in silico*, and it is given by the following:

$${\text{NI}}^{\left(i\right)}=\frac{{\text{BNI}}_{\text{pre}}-{\text{BNI}}_{\text{post}}^{\left(i\right)}}{{\text{BNI}}_{\text{pre}}},$$

where ${\text{BNI}}_{\text{pre}}$ is the BNI before node removal, and ${\text{BNI}}_{\text{post}}^{\left(i\right)}$ is the BNI after the removal of node i. Following previous studies, we selected the parameter K such that ${\text{BNI}}_{\text{pre}}=0.5$ (Goodfellow et al., 2016; Lopes et al., 2017, 2019, 2020). ${\text{BNI}}_{\text{post}}^{\left(i\right)}$ is typically smaller than ${\text{BNI}}_{\text{pre}}$, meaning that ${\text{NI}}^{\left(i\right)}$ is usually positive. Thus, the ${\text{NI}}^{\left(i\right)}$ ranges typically between 0 and 1, where 0 corresponds to a node removal that has no effect on seizure generation ( ${\text{BNI}}_{\text{post}}^{\left(i\right)}={\text{BNI}}_{\text{pre}}$), and 1 corresponds to a node removal that stops seizures in the network ( ${\text{BNI}}_{\text{post}}^{\left(i\right)}=0$). The higher the NI, the more important the node is for seizure dynamics. As above, we also took the mean NI values of each node across the three functional networks per individual for the statistical analysis presented below.

Figure 1 summarizes the key steps of our methods.

### Statistical methods

We used a one-sided Mann–Whitney *U* test to assess whether the mean BNI values were higher in the PPR group than in the non-PPR group. We also used the same test to compare the mean NI in the occipital areas of the two groups. Furthermore, we performed an exploratory analysis where we used the two-sided Mann–Whitney *U* test to evaluate whether the mean NI of each brain area (other than the occipital areas) was different between the two groups. To correct for multiple comparisons within each family of tests, we applied the Bonferroni–Holm procedure to each of these three analyses separately. For all of these Mann–Whitney *U* tests, we report the *U*-statistic and *z* score. We further quantified statistical differences by using estimation statistics (Ho et al., 2019). We used the median difference as the effect size and 95% confidence intervals (CIs) were built using 5000 bootstrap samples. To measure these estimation statistics, we used the tools provided by (Ho et al., 2019; https://www.estimationstats.com).

In the case of statistically significant results, we further quantified the difference between the two groups using the receiver operating characteristic (ROC) curve, area under the curve (AUC), and sensitivity and specificity.

### Data availability

MATLAB scripts implementing the methods described in the article are freely available online at https://github.com/ml0pe5/Photostimulation_BNI_NI.

## Results

**Figure 2.**
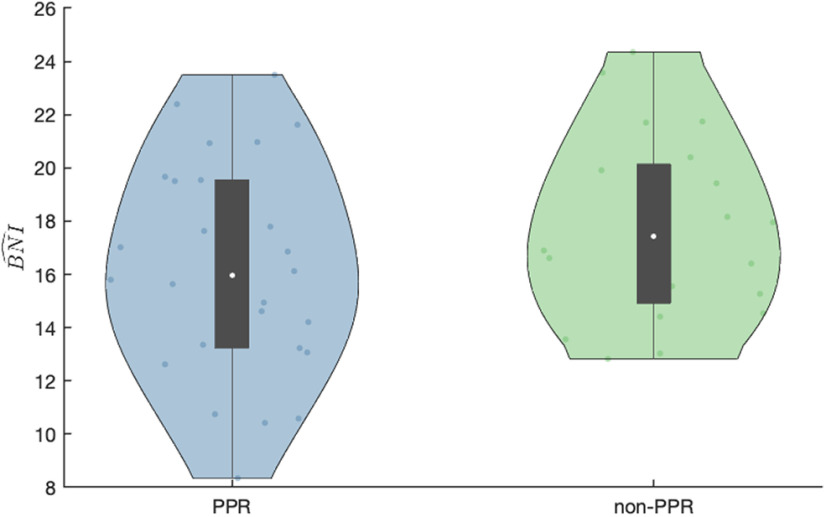
Violin plot of BNI comparing the PPR and non-PPR groups. The white dot represents the median, the black box represents the interquartile range, and the other dots within the shaded region correspond to the BNI of each individual. The BNI values are not statistically higher in the PPR group than in the non-PPR group (*p* = 0.89, one-sided Mann–Whitney *U* test).

Do people with epilepsy who had a PPR to IPS have higher widespread excitability than people who did not have a PPR to IPS? Assuming that they do have a higher widespread excitability, does it translate into a higher propensity to seizures as assessed from their resting-state functional networks? To address this latter question, we measured the BNI of each individual from interictal EEG recordings before the IPS study. Figure 2 shows the BNI of the two groups: the PPR group (i.e., positive PPR on IPS) and the non-PPR group (i.e., no PPR on IPS). We found that the BNI is not statistically significantly higher in the PPR group ( p=0.89; one-sided Mann–Whitney *U* test, U=556, z=−1.23). We further found that the unpaired median difference of BNI was −1.48 with a 95% confidence interval equal to –4.64 to 1.40. These results suggest that PPRs are not a consequence of an individual having a higher propensity to generate seizures (i.e., a lower threshold for seizure emergence).

**Figure 3.**
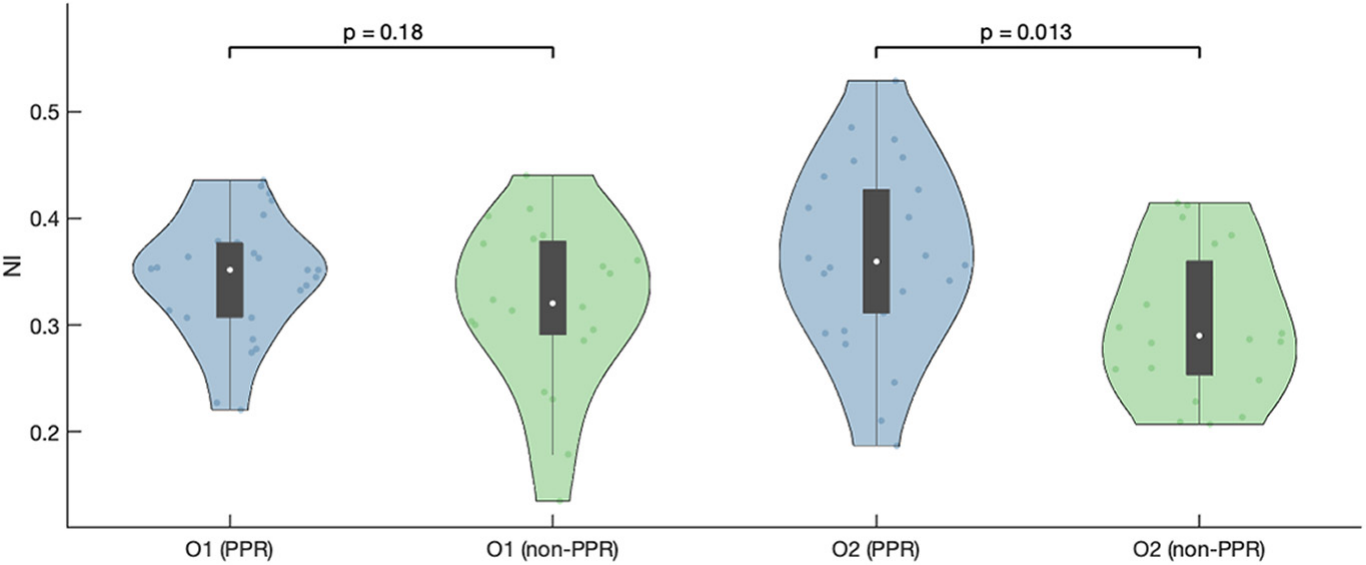
NI of the occipital lobe nodes in the PPR and non-PPR groups. The NI values in the O2 region are statistically significantly higher in the PPR group than in the non-PPR group. The *p*-values correspond to one-sided Mann–Whitney *U* tests corrected with the Bonferroni–Holm procedure.

**Figure 4.**
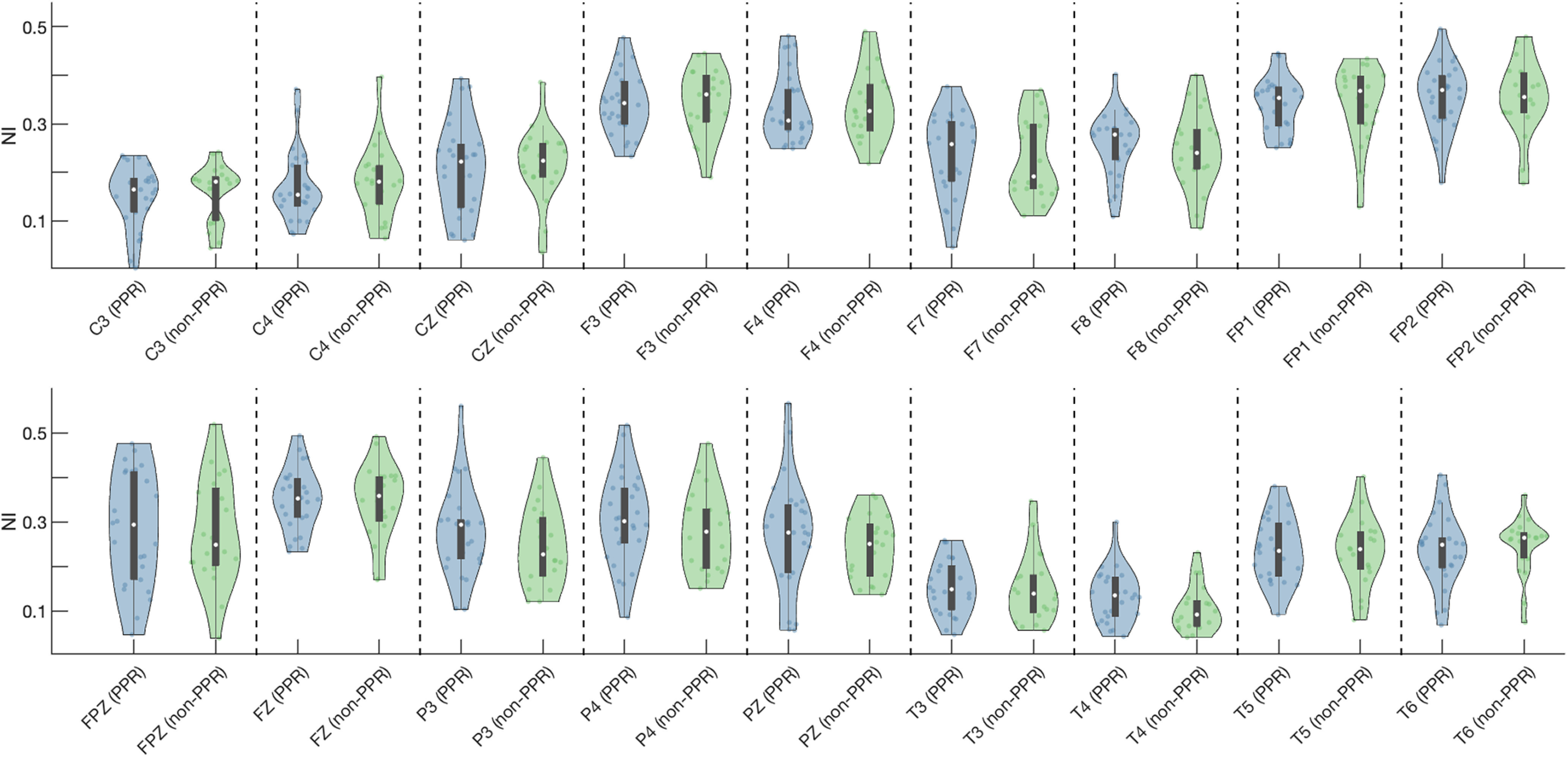
NI of all the network nodes in the PPR and non-PPR groups. This figure excludes the nodes corresponding to the occipital lobe nodes (presented in Fig. 3). *p*-Values, effect sizes, and confidence intervals are presented in Table 3. All these differences in NI are not statistically significant.

Having observed that the PPR group is not more prone to seizures than the non-PPR group, we hypothesized that their paroxysmal response to light stimulation results from a higher ictogenic propensity in the occipital lobe compared with the non-PPR group. Thus, we tested whether the occipital areas of PPR individuals had a stronger contribution to seizure generation within the whole brain network than they did in non-PPR individuals. Figure 3 compares the NI in the occipital areas (channels O1 and O2) between the PPR and non-PPR groups. We observed that the median of the NI of the O1 region was higher in the PPR group relative to the non-PPR group, but it was not significant ( p=0.18; one-sided Mann–Whitney *U* test, corrected for multiple comparisons, U=652, z=0.90). We found a significantly higher NI in the O2 region in the PPR group relative to the non-PPR group ( p=0.013; one-sided Mann–Whitney *U* test, corrected for multiple comparisons, U=724, z=2.49). Estimation statistics further supported these observations: for O1, the unpaired median difference of NI was 0.03 with a 95% confidence interval of −0.02 to 0.06; and for O2 the effect size was 0.07 with a 95% confidence interval of 0.01–0.12. As an exploratory analysis, we also tested whether any other region had a different ictogenicity in the PPR group compared with the non-PPR group, and we found that no region had a statistically different NI between the two groups (Fig. 4, Table 3).

**Table 3:** Assessment of NI differences between the two groups in each node (as presented in **Fig. 4)**

Node	Uncorrected *p*-value	Corrected *p*-value	*U* statistic	*z*	Effect size	CI lower limit	CI upper limit
C3	0.54	1	583	−0.6	−0.016	−0.048	0.025
C4	0.35	1	568	−0.94	−0.026	−0.060	0.011
CZ	0.59	1	586	−0.54	−0.002	−0.057	0.046
F3	0.65	1	590	−0.45	−0.019	−0.068	0.029
F4	0.73	1	595	−0.34	−0.020	−0.072	0.031
F7	0.63	1	633	0.48	0.068	−0.024	0.124
F8	0.56	1	638	0.59	0.038	−0.014	0.073
FP1	0.47	1	578	−0.72	−0.014	−0.064	0.050
FP2	0.94	1	607	−0.08	0.015	−0.027	0.058
FPZ	0.75	1	626	0.32	0.045	−0.072	0.179
FZ	0.79	1	599	−0.25	−0.006	−0.056	0.050
P3	0.29	1	659	1.05	0.067	−0.001	0.121
P4	0.35	1	653	0.92	0.022	−0.045	0.106
PZ	0.29	1	659	1.05	0.024	−0.032	0.096
T3	0.60	1	635	0.52	0.010	−0.046	0.051
T4	0.08	1	689	1.72	0.042	−0.002	0.079
T5	0.85	1	602	−0.19	−0.004	−0.057	0.057
T6	0.26	1	560	−1.12	−0.015	−0.051	0.031

The *p*-values, *U* statistics, and *z*-scores correspond to two-sided Mann–Whitney *U* tests assessing whether NI is different between the two groups at a given node. The *p*-values were corrected using the Bonferroni–Holm procedure, and none is significant. The effect size (median difference of NI) and confidence intervals further show that there are no statistical differences between the groups in any of these nodes.

Given the observation that the O2 was the most different area in terms of NI between the two groups, we further measured its ability to classify the two groups. We calculated the ROC curve and found an AUC of 0.72, a sensitivity of 0.85, and a specificity of 0.55.

To better characterize our findings, we performed a supplementary analysis. Considering that previous studies have suggested that photosensitive epilepsy may be differentiated from other types of epilepsy by analyzing resting-state EEG power spectrum (Vaudano et al., 2017) or by analyzing resting-state EEG functional networks (Varotto et al., 2012), we tested whether such types of analyses could distinguish our two groups of individuals. Our purpose was to determine whether our findings could be explained by these straightforward spectral or connectivity analyses of EEG signals. Based on the study by Vaudano et al. (2017), we computed the relative power in the alpha band in the occipital electrodes (O1 and O2) and tested whether the relative power was higher in the PPR group than in the non-PPR group. We observed no statistical differences (Table 4). Furthermore, we tested whether the occipital electrodes had higher connectivity strength in the PPR group relative to the non-PPR group (Rubinov and Sporns, 2010). Again, we found no statistical differences (Table 4).

**Table 4:** Assessment of relative power and connectivity differences on occipital electrodes between the PPR and non-PPR groups

Measure	Node	Uncorrected *p*-value	*U* statistic	*z*-score	Effect size	CI lower limit	CI upper limit
Relative low alpha power	O1	0.79	575	−0.81	−0.028	−0.095	0.047
	O2	0.74	582	−0.65	−0.013	−0.089	0.049
Relative alpha power	O1	0.86	562	−1.10	−0.078	−0.13	0.049
	O2	0.80	573	−0.85	−0.039	−0.14	0.041
Occipital connectivity	O1	0.94	543	−1.52	−0.76	−2.18	0.48
	O2	0.76	580	−0.70	−0.34	−2.18	1.17

The relative power was computed following the methods of Vaudano et al. (2017). We considered the following two frequency bands: low alpha power (6–9 Hz) as in the main analysis; and alpha power (7.5–12.5 Hz) as in the study by Vaudano et al. (2017). The occipital connectivity corresponds to the connection strength of the electrodes (i.e., sum of in-strength and out-strength; Rubinov and Sporns, 2010). The *p*-values, *U* statistics, and *z*-scores correspond to one-sided Mann–Whitney *U* tests assessing whether the relative power (or connectivity strength) is higher in the PPR group relative to the non-PPR group at a given occipital electrode. All *p*-values are not significant. The effect size (median difference) and confidence intervals further show that there are no statistical differences between the groups when using these measures.

## Discussion

In this study, we consider the hypothesis that PSE may be underpinned by both occipital and more widespread cortical hyperexcitability (Padmanaban et al., 2019). We assess whether such hyperexcitability is an enduring feature inferable from resting brain activity of people with PSE. We used interictal scalp EEG recordings from two groups of individuals with IGE, one that had PPR during IPS, and another that did not have PPR. We inferred functional networks from the EEG data and studied the propensity of the participants’ brain networks to generate seizure activity using a computer model. By simulating the emergence of seizure-like dynamics on the networks, we were able to compare the overall ictogenic propensity and local ictogenic propensity across individuals.

We first tested whether the PPR group had a higher BNI than the non-PPR group. A higher BNI would suggest a higher propensity of the PPR group to have seizures, which could be indicative of widespread hyperexcitability in this group. We found that the BNI was not statistically different between the two groups. While this result suggests that there is not an enduring hyperexcitability identifiable from interictal EEG data, we acknowledge that there is an alternative interpretation. Namely, the BNI may better reflect the expectable seizure frequency of an individual (Lopes et al., 2021) rather than their hypothesized widespread hyperexcitability. From this perspective, the BNI was not expected to be higher in the PPR group relative to the non-PPR group, as observed, because people with PSE do not have higher seizure rates than people without PSE (Covanis, 2005; Verrotti et al., 2012).

We then tested whether the PPR group had a higher occipital NI than the non-PPR group. We found that indeed the NI is higher in occipital regions of the PPR group relative to the non-PPR group, in particular in the right occipital region (O2). This result suggests that there is an enduring hyperexcitability of the occipital regions in PSE, and that this hyperexcitability can be inferred even from apparently normal scalp EEG recordings, without requiring the use of stimulation. More specifically, a higher NI of a region indicates that this region is more relevant to seizure emergence. Note, however, that we do find other regions with higher NI than the occipital lobe (e.g., the frontal areas, FP1 and FP2; Fig. 4). Together, these results suggest that people with PSE have a higher ictogenic propensity in their occipital lobe compared with other individuals with epilepsy, but that this region is not their main driver of seizures (in line with people without PSE). We further showed that, using NI to classify people with PSE, we obtained a sensitivity of 0.85 and a specificity of 0.55. Our results are in line with previous evidence that has indicated occipital hyperexcitability in people with PSE (Wilkins, 1995; Porciatti et al., 2000; Ferlazzo et al., 2005; Padmanaban et al., 2019).

There have been previous studies assessing PSE based on resting-state EEG functional connectivity. Varotto et al. (2012) computed partial directed coherence from 10 individuals with PSE and 10 healthy control subjects. They found that people with PSE had enhanced connectivity, predominantly involving the anterior cortical regions. A key advantage of our study compared with this one is that we compared two groups of people with epilepsy, one with PSE and one without, allowing us to assess features that should be specific to PSE, rather than of epilepsy more generally. Vaudano et al. (2017) analyzed resting-state EEG-fMRI from 16 individuals with genetic generalized epilepsy with PSE, 13 individuals with genetic generalized epilepsy without PSE, and 15 individuals with focal epilepsy. They found that the PSE group had significantly higher mean alpha power than the other two groups. Based on both EEG and fMRI, they showed that the “cortical–subcortical network generating the alpha oscillation at rest is different in people with epilepsy and visual sensitivity.” While not having fMRI in our dataset, our analysis went beyond those of these two studies. Rather than focusing on differences in network structure between groups, we placed a model of ictogenicity on the networks to pose hypotheses about how their structure may influence ictogenicity. Additionally, we tested whether we could distinguish our two groups of individuals based on analyses of alpha power and connectivity strength, similar (but not equivalent) to those performed by Vaudano et al. (2017) and Varotto et al. (2012). We found that those measures could not differentiate our groups of individuals, which further supports the use of our computational method. Nevertheless, we note that our analyses of EEG spectrum and connectivity are not replications of the analyses reported by Vaudano et al. (2017) and Varotto et al. (2012). In addition to some methodological differences, both the data and cohorts of individuals were not directly comparable to ours. Vaudano et al. (2017) used 32-channel EEG and fMRI, while we used 20-channel EEG. Varotto et al. (2012) compared individuals with PSE with healthy control subjects, whereas we assessed a cohort of IGE patients with and without PSE. Our purpose was not to replicate their studies, but rather to assess whether we could differentiate our two groups of individuals using straightforward EEG spectrum or connectivity analyses, which to some extent underlie our computational framework. Future work should aim to replicate together our study and their studies to better assess whether the results are concordant or complementary.

In conclusion, we applied the BNI and NI frameworks to interictal scalp EEGs from people with epilepsy, and we found that people with PSE have higher NI in occipital regions than people without PSE. Our findings suggest that the mechanisms of PSE may be revealed by enduring features present in interictal brain states. Our results also suggest that the NI applied to resting-state EEG may aid the diagnosis of PSE without the need of stimulation.

## Synthesis

Reviewing Editor: William Stacey, University of Michigan Decisions are customarily a result of the Reviewing Editor and the peer reviewers coming together and discussing their recommendations until a consensus is reached. When revisions are invited, a fact-based synthesis statement explaining their decision and outlining what is needed to prepare a revision will be listed below. The following reviewer(s) agreed to reveal their identity: Sebastien Naze, Piotr Slowinski.

The reviewers and I have discussed the paper and find it to be an interesting analysis with interesting result, but there are several questions we would like addressed. Most importantly, we would like to see an additional analysis on the data that would help determine if there were “standard” differences between the two groups’ EEGs, which might help explain or motivate the results of your model (#4 below).

1. the statistical analysis appears to be inconsistent. First, it seems only the T4 electrode was subjected to multiple comparisons; that correction must be applied to EVERY analysis, not just the ones that disagree with the hypothesis. We recommend having a table with all p values, corrected p values, and to maintain the violin plots. For instance, l. 257: Since a multiple comparison correction is applied on Fig 3-1, why not also reporting the corrected p-value of the O1 and O2 electrodes in Fig 3 for consistency? All electrodes should have the same analysis, not just those in 3-1. The data in 3-1 should be part of the main paper, not “extended data.”

2. Of note, eNeuro prefers estimation statistics rather than p-values, and this paper is a prime example of why-it is better to show the data and have the reader determine what it means. (Estimation Statistics, One Year Later | eNeuro)

3. If Bonferroni correction is too stringent, FDR or FWE could be an option.

4. The analysis is somewhat superficial-the results are presented but there is little insight as to why this effect occurs. That should be improved. For instance, as mentioned in the discussion, previous studies from Varotto et al. (2012) and Vaudano et al. (2017) find differences in coherence and power-spectra. As presented, we do not know if the EEGs have those differences in your dataset; that is a very important piece of information. We recommend adding an analysis such as those two papers (or others) on the same data to determine if there are any raw signal characteristics that distinguish the photosensitive patients. The goal should be to determine if there are inherent differences in the EEGs themselves between the two groups. Either answer is useful: either there is a difference, which might explain your findings, or there is not, which would give strength to using your method to find hidden differences. Therefore, even no significant difference in the analysis between groups would further encourage the use of the model.

Minor comments:

l.166: on the theta-model: any background besides Lopes work? Original authors of the model?

l.218-220: NI(i) takes values between [0, 1]. From the equation though, when BNI_post > BNI_pre then NI(i) should take negative values. This case would indicate that the node prevents seizure, is this case never considered?

l.242: later à latter

## Author Response

We thank the editors and reviewers for their encouraging review and useful comments.

Please find our response below to each comment.

Computational Neuroscience Model Code Accessibility Comments for Author (Required):

The link to the code is hidden, but must be evaluated by the reviewers. Is there a way to anonymize it? This really would be preferable. If not, you can provide the link for the editor to view separately.

We have made an anonymous account in GitHub and made available a copy of the code anonymised: https://github.com/anonym0us001/Photostimulation_BNI_NI-anonymized

Synthesis Statement for Author (Required):

The reviewers and I have discussed the paper and find it to be an interesting analysis with interesting result, but there are several questions we would like addressed. Most importantly, we would like to see an additional analysis on the data that would help determine if there were “standard” differences between the two groups’ EEGs, which might help explain or motivate the results of your model (#4 below). This will require some additional work, but hopefully can be completed within the 2 months’ time frame.

We thank the reviewers and editors for their review and suggestions. We addressed the specific suggestions below.

1. the statistical analysis appears to be inconsistent. First, it seems only the T4 electrode was subjected to multiple comparisons; that correction must be applied to EVERY analysis, not just the ones that disagree with the hypothesis. We recommend having a table with all p values, corrected p values, and to maintain the violin plots. For instance, l. 257: Since a multiple comparison correction is applied on Fig 3-1, why not also reporting the corrected p-value of the O1 and O2 electrodes in Fig 3 for consistency? All electrodes should have the same analysis, not just those in 3-1. The data in 3-1 should be part of the main paper, not “extended data.”

Testing hypotheses for electrodes O1 and O2 is different from testing hypotheses for the other electrodes. While we did expect to find higher NI in electrodes O1 and/or O2, we did not have such expectation for the other electrodes. Multiple comparison corrections are supposed to be applied to control for family-wise errors. O1 and O2 belong to the same family of tests (our test hypothesis: occipital lobe has higher NI in the PPR group), while the other electrodes belong to a separate family of tests (exploratory analysis: does any other electrode show NI differences between the groups). As an intuitive rationale for this, it would be illogical that our results on testing the initial hypothesis about the occipital lobe would be confounded by additional analyses on other areas. If we would apply multiple comparisons to all electrodes, this would also imply that if our analyses would comprise, say, 64 electrodes instead of 20, the chances of us finding a statistically significant result would be compromised (leading to a risk of type II error). Thus, we agree that corrections must be applied to every analysis, but it is necessary to acknowledge that not all analyses test the same family of hypotheses. In our previous analysis, we had overlooked the application of correction to the family comprising O1 and O2 which implies that the threshold of significance is lowered by a factor of 2 for these two electrodes when using Bonferroni correction. In contrast, the application of Bonferroni correction to the family comprising the other 18 electrodes means that the threshold of significance is reduced by a factor of 18 for those electrodes. In our analysis, we used the Bonferroni-Holm procedure which is slightly less conservative than the Bonferroni correction. In the revised manuscript, we have also applied the Bonferroni-Holm procedure to the family comprising the O1 and O2 electrodes.

Following the suggestions, we have added a table with all p-values (table 3), effect sizes and confidence intervals for the exploratory analysis of NI. Concerning BNI and NI analysis in the occipital lobe, we reported the p-values, effect sizes, and confidence intervals in the text. We have also moved Figure 3-1 to the main paper (now Figure 4). Furthermore, we updated section 2.5 to better describe the exploratory analysis, to clarify the application of the Bonferroni-Holm procedure, and to report the additional statistical methods that were used.

2. Of note, eNeuro prefers estimation statistics rather than p-values, and this paper is a prime example of why-it is better to show the data and have the reader determine what it means. (Estimation Statistics, One Year Later | eNeuro)

We thank the reviewers and editor for the suggestion. As mentioned above, we have now reported effect sizes and confidence intervals for all our analyses in the revised manuscript.

3. If Bonferroni correction is too stringent, FDR or FWE could be an option.

We have kept the Bonferroni-Holm procedure. (Other corrections would provide the same conclusions in terms of statistical significance.)

4. The analysis is somewhat superficial-the results are presented but there is little insight as to why this effect occurs. That should be improved. For instance, as mentioned in the discussion, previous studies from Varotto et al. (2012) and Vaudano et al. (2017) find differences in coherence and power-spectra. As presented, we do not know if the EEGs have those differences in your dataset; that is a very important piece of information. We recommend adding an analysis such as those two papers (or others) on the same data to determine if there are any raw signal characteristics that distinguish the photosensitive patients. The goal should be to determine if there are inherent differences in the EEGs themselves between the two groups. Either answer is useful: either there is a difference, which might explain your findings, or there is not, which would give strength to using your method to find hidden differences. Therefore, even no significant difference in the analysis between groups would further encourage the use of the model.

We thank the editor and reviewers for their very valuable suggestion. We have now added an additional analysis based on power spectrum and connectivity similar to those performed by Varotto et al. and Vaudano et al. to the manuscript (see last paragraph of the Results and Table 4). We found that these measures did not differ between our two groups of patients, further encouraging the use of our computational model. We also updated the Discussion to comment on this.

Minor comments:

l.166: on the theta-model: any background besides Lopes work? Original authors of the model?

We added the citation to the original work on the theta model (Ermentrout and Kopell, 1986), and updated the References section accordingly.

l.218-220: NI(i) takes values between [0, 1]. From the equation though, when BNI_post > BNI_pre then NI(i) should take negative values. This case would indicate that the node prevents seizure, is this case never considered?

The reviewers are correct, NI(i) can take values from -1 to 1. However, as we had written in the manuscript, it typically takes values from 0 to 1, i.e., it is very uncommon for the NI(i) to be negative, at least in the context of our analysis (as it can be seen in the figures). To clarify this in the manuscript, we added the following text:

"BNI_post^((i) ) is typically smaller than BNI_pre, meaning that NI^((i) ) is usually positive. Thus, the NI^((i) ) ranges typically between 0 and 1 [...]"

l.242: later à latter

We have corrected this typo.
